# Influence of Moisture and Tool Temperature on the Maximum Stretch and Process Stability in High-Speed 3D Paper Forming

**DOI:** 10.3390/ma18122894

**Published:** 2025-06-18

**Authors:** Heike Stotz, Matthias Klauser, Johannes Rauschnabel, Marek Hauptmann

**Affiliations:** 1 Independent Researcher, 70186 Stuttgart, Germany; 2 Independent Researcher, 71254 Ditzingen, Germany; 3 Syntegon Technology GmbH, 71332 Waiblingen, Germany; 4 Fraunhofer IVV, 01189 Dresden, Germany

**Keywords:** three-dimensional paper forming, moisture preconditioning, process optimization

## Abstract

This study investigates how moisture preconditioning and thermal parameters affect the stretchability of paper in 3D forming, with the goal of extending geometric forming limits and enhancing process stability. Multidimensional tensile tests were performed on FibreForm Duo (310 g/m^2^) using a hemispherical punch. Key variables included water bath dwell time, punch temperature, and contact time, simulating industrial conditions in high-speed packaging. A short duration of water bath immersion (1–3 s) led to rapid moisture uptake (−20%), resulting in significantly improved formability. Compared to unconditioned samples, the maximum stretch increased by up to 3.5 percentage points. The process window identified (3.03 s dwell time; 70 °C punch temperature; 1.08 s contact time to punch) yielded a predicted stretch of 16.5%, representing a notable expansion of the material’s geometric forming capacity. Regression analysis (R^2^ = 0.8946) confirmed the strong statistical significance of all parameters.

## 1. Introduction

**Figure 1:**
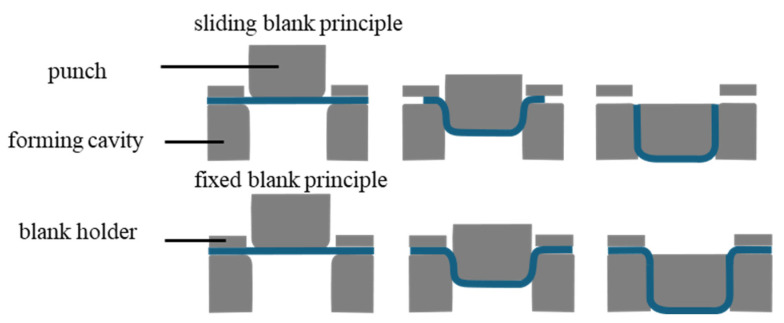
Schematic representation of the sliding blank principle and fixed blank principle [1].

The production of three-dimensionally molded paper containers is based on two principles—the ‘sliding blank principle’ and the ‘fixed blank principle’. With the ‘sliding blank principle’, the paper blank is pulled into the mold cavity, creating folds. These can be controlled by different variables. With the ‘fixed blank principle’, the cardboard blank is clamped. The stretchability and strength of the paper are important in the ‘fixed blank principle’, while the compression properties of the paper play a role in the ‘sliding blank principle’. The two principles are shown schematically in Figure 1 [1].

**Figure 2:**
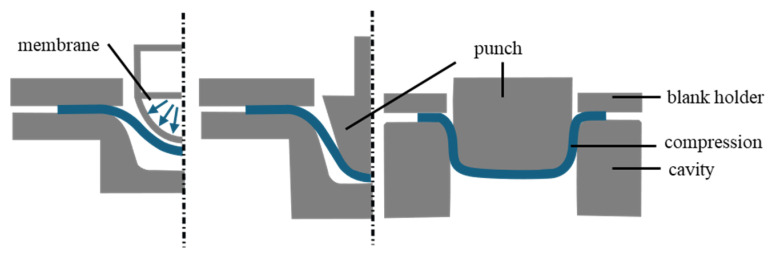
Schematic representation of hydroforming, press forming, and deep drawing [1].

Paper forming processes with higher degrees of deformation can be divided into press forming, hydroforming, and deep drawing. The schematic principles are shown in Figure 2.

The press forming process uses a punch, die, and blank holder to shape pre-cut cardboard blanks. The blank holder controls wrinkle formation as the blank is pressed into the cavity. If a polymer layer is present, it softens and seals the wrinkles during the process. The flange is also flattened by the blank holder [2]. Springback occurs after removing the formed part, with forming degrees between 0.3 and 0.4 and cone angles of 10° to 30° [1,3].

In hydroforming, an incompressible fluid, enclosed by a rubber membrane, shapes the material into the die. Clamps or wrinkle holders secure the material, while the membrane applies pressure to maximize stretch and prevent wrinkles. The current drawing ratio is around 0.2 [4].

In deep drawing, the punch and die are heated, and the punch presses the material into the die. Blank holders are used to fix the material and control wrinkling. The formed part can either be pulled out in the stroke direction, creating a sealing edge, or pushed through the die, without an edge for sealing. Unlike press forming, the blank is immediately compressed between the punch and cavity as the gap is smaller than the material’s thickness. A conical punch shape helps reduce cracks by counteracting material thickening. The cavity wall must be at a 90° angle. Springback occurs after the removal of the cup. [1,4].

Deep drawing, press forming, and hydroforming typically employ heated metal tools at temperatures between 140 °C and 180 °C. However, due to the high forming speed, the temperature of the paper itself often does not exceed 100 °C [4]. Additionally, a moisture content in the range of 10% to 20% has been shown to improve the forming results. Specimens were either conditioned in a climate chamber [5] or moistened using experimental setups like rolling or spray moistening [6]. One key finding was that similar forming results could be achieved with samples moistened by rolling penetration with less moisture compared to those conditioned in the climate chamber, which had a higher total water content [4]. Furthermore, spray-moistened samples exhibited a better formability than those preconditioned in a climate chamber [5].

Many goods, especially in the food sector, are packaged using form–fill–seal technology. This process involves forming, filling, and sealing the packaging material within a single machine, ensuring high production efficiency and reliability [7]. There are two main methods of material feeding—from a roll or individual blanks. Direct drawing from the roll often causes cracks or delamination due to the limited extensibility of the material [8]. As this process is widely used in mechanical engineering, especially for various packaging processes including plastic materials, an investigation into improving the stretchability of the paper is very useful.

A higher humidity increases the paper moisture content, improving extensibility while reducing tensile strength and modulus. Water in the fiber network weakens bonds, encouraging plastic deformation and lowering network strength [9]. Temperature has a similar effect, softening the fiber structure, especially in relation to hemicellulose and lignin [10]. Both temperature and humidity impact paper by altering its moisture content, softening fiber wall polymers, and affecting the glass transition [10]. The softening temperatures of wood polymers vary with moisture, as water reduces fiber stiffness [11]. In dry conditions, cellulose, lignin, and hemicellulose soften at 230 °C, 205 °C, and 180 °C, respectively, but these temperatures drop significantly at 6% moisture, typical for air-dried paper [11]. Moisture-sensitive cellulose and hemicellulose soften with increasing temperature and humidity, while crystalline cellulose remains stable until about 240 °C [12]. However, lignin is less affected by moisture but softens at higher temperatures. Water acts as a plasticizer, reducing fiber stiffness by weakening hydrogen bonds, enabling deformation [13]. Moisture has a stronger impact on paper extensibility than temperature alone [10]. Nevertheless, the effects of moisture and heat cannot easily be observed separately [14]. The aim of this study is to investigate multidimensional tensile tests with preconditioning to observe the effect of material moisture, punch temperature, and cycle time on the maximum stretch of the material.

In high-speed packaging lines with cycle times ranging from 1 to 3 s, the fast and controlled moistening of the paper is crucial to ensure reproducible and high-quality forming. Franke et al. (2018) demonstrated that both increased moisture and elevated temperatures significantly enhance the formability of paperboard materials. Steam-assisted forming was shown to reduce springback and effectively control wrinkling. However, the moisture absorption process is time-consuming, e.g., dry samples required up to 14 s to reach sufficient moisture levels [15].

Stotz et al. (2022) investigated various moistening techniques in this context and highlighted water bath moistening as being particularly advantageous. In an in-line setup, this method achieved rapid and homogeneous moisture distribution within just 1.6 to 4 s. Unlike other approaches discussed in the study, it enabled the consistent conditioning of the paper prior to subsequent heating (to 100 °C or 200 °C) and forming. Their findings confirmed that higher moisture content and temperature substantially improve the material’s forming behavior [16].

The primary objective of this study is to enhance the formability of paper in 3D forming processes while simultaneously ensuring process stability under fluctuating moisture conditions. These fluctuations arise from the hygroscopic nature of paper, which continuously exchanges moisture with the surrounding air until it reaches a temperature- and humidity-dependent equilibrium. In non-conditioned production environments, this can lead to significant moisture variations—typically ranging from 4–5% absolute moisture at 20 °C and 30% relative humidity in winter to 8–9% at 30 °C and 50% relative humidity in summer [17].

To address this challenge, the present work builds on the promising results of Stotz et al. (2022) [16], who demonstrated improved formability using a water bath in combination with conduction-based preheating—without requiring a heated punch. This study investigates whether a comparable improvement in formability can be achieved by directly applying thermal energy through a heated punch instead. If successful, this would allow for the elimination of one process station, thus simplifying the system layout and reducing energy and maintenance requirements.

## 2. Materials and Methods

The paper material used is FibreForm Duo (BillerudKorsnäs, Solna, EU, Sweden & KAPAG Karton + Papier AG, Muhen, EU, Switzerland), with a grammage of 310 g/m^2^. It consists of two layers of 150 gsm FibreForm (BillerudKorsnäs) that are glued (20 g/m^2^) together. This uncoated bleached chemical cellulose carton has a maximum stretch of 15% in the machine direction and 10% in the cross direction, as specified by the manufacturer according to ISO 1924-3 [18,19]. The high maximum stretch values can be attributed, in part, to the fact that the paper is creped. Creped paper, unlike uncreped paper, undergoes a compaction process that introduces micro-compressions and micro-creping, increasing its elongation potential, especially in the machine direction (MD), by deforming the network and fibers [13,20]. Conventional paper exhibits a maximum stretch from 2% to 5%. The cross-direction (CD) elongation is generally higher than the machine direction (MD) elongation due to the lower mechanical resistance of the material in the CD [13]. The high stretch values of the paper used are highly beneficial for paper forming applications.

**Figure 3:**
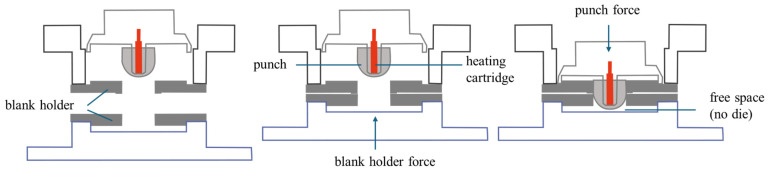
Schematic drawing of tooling.

The multidimensional tensile tests were conducted on a test rig at Syntegon technology GmbH. The forming unit consisted of a servo-driven punch, a die, and a mask. During a forming cycle, the die moved towards the mask, fixed the paper, and provided the blank holder force. The punch then pressed the fixed paper into the mold with a defined force. After forming, the punch and die returned to their starting positions. The punch can be heated by means of an integrated heating cartridge. A schematic drawing of the tooling is shown in Figure 3.

The punch is hemispherical in shape, while the die is designed as a simple hole. The punch stroke can be adjusted to the maximum extensibility at which no cracks occur in the formed cavity. The maximum punch stroke is validated using a total of five samples. The trials are conducted according to the experimental design outlined in Table 1. The stretch is calculated according to the stroke of the punch and the change in the length of the paper specimen from the unformed to the formed state.

**Figure 4:**
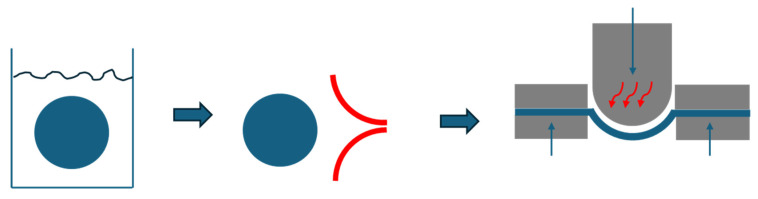
Schematic process of forming trials: immersion in water bath; removal of surface water by rubber lips; multidimensional tensile tests.

The paper samples were cut into circular shapes with a diameter of 76 mm. Each sample was fully immersed in a water bath using tongs for the duration specified in the test plan. After moistening, the excess water was removed. Therefore, the sample was pushed between two touching rubber lips. Multidimensional tensile tests were conducted using a hemispherical punch. The punch temperature and cycle time were adjusted for each run according to the test plan. The sample was manually placed in the forming station and the molding process was started via HMI (Human–Machine interface) with the respective parameter settings. The forming process was carried out with a blank holder force of 20 kN and a punch force of 10 kN, ensuring the paper blank was completely fixed between the die and the blank holder. The sample was examined for cracks, and the stroke of the punch was adjusted accordingly. The maximum stretch achieved was validated using 5 samples. The schematic process is shown in Figure 4.

The moisture levels of the paper samples were assessed using a Halogen Moisture Analyzer HR73 (Mettler-Toledo GmbH, Gießen, EU, Germany). Each wetted sample was placed on an aluminum dish within the analyzer, and the initial weight of the sample was recorded. The drying process continued until no further weight loss was detected. The temperature was maintained at 105 °C in accordance with DIN EN ISO 287 [21]. The moisture content of the paper specimen before forming was measured in individual runs in accordance with the settings outlined in the test plan in Table 1. The moisture content after forming was measured immediately after removing the formed paper specimen from the test rig. The surface of the paper that is in contact with the punch was calculated with respect to the maximum stretch reached by each test setting. The difference in moisture content between the various parameter settings was then related to this contact area. This conversion yields the moisture differences resulting from the conductive drying caused by the punch.

In this study, forming trials were conducted using a structured experimental design, as shown in Table 1, that varied three key factors—dwell time in the water bath, punch temperature, and punch contact time. The dwell time in the water bath was tested at five levels—0, 1, 2, 3, and 4 s. A dwell time of 0 s served as a reference point with no moistening, while 1 s simulated realistic conditions for in-line moistening stations in industrial processes—corresponding to humidification times of approximately 0.5 to 1.5 s at machine speeds of 30 to 40 cycles per minute. The longer times (2 to 4 s) allowed for the examination of saturation effects. The punch temperature was tested at setpoints of 70 °C, 95 °C, and 120 °C, corresponding to measured temperatures of approximately 64.5 °C, 86.5 °C, and 109.8 °C on the punch. The selected temperature levels were chosen to investigate a range spanning both below and above the boiling point of water. Based on the literature, which suggesting an improved extensibility at 60–70 °C [13], the starting temperature was set to 70 °C. Tooling temperatures exceeding the boiling point are relevant in 3D paper forming, particularly in fixed blank principle technologies (140–180 °C) [4]. However, while these fixed blank technologies rely on a combination of paper extensibility, compressibility, and friction, the present study employs the fixed blank principle, where drying significantly influences the forming result. Therefore, the upper temperature was reduced to 120 °C to better reflect the drying-dominated mechanism of the fixed blank process used here. Punch contact time was indirectly varied by adjusting the cycle rate of the forming unit. Two levels were used—10 cycles per minute, corresponding to a long contact time of 4.332 s, and 40 cycles per minute, representing a short contact time of 1.083 s. This allowed the investigation of time-dependent heat transfer and moisture loss during forming. The number of experimental units per parameter setting was n= 30. The number of specimens tested per parameter setting was I = 3.

## 3. Results and Discussion

In Figure 2, the diagram on the left illustrates the deformation geometry during the forming process with a heated punch. The un-deformed initial length is labeled l_0_, which spans horizontally before forming begins. As the punch descends by a defined stroke height (“Stroke Punch”), the material undergoes stretching and bending, resulting in the following two distinct path segments in the deformed state:

- l_1-a_ (green) corresponds to a straight region of the formed contour, which experiences moderate deformation.
- l_1-b_ follows a curved trajectory, representing the area of greatest deformation. This section is subjected to the highest local strain and represents the most strongly formed zone of the material.

**Figure 5:**
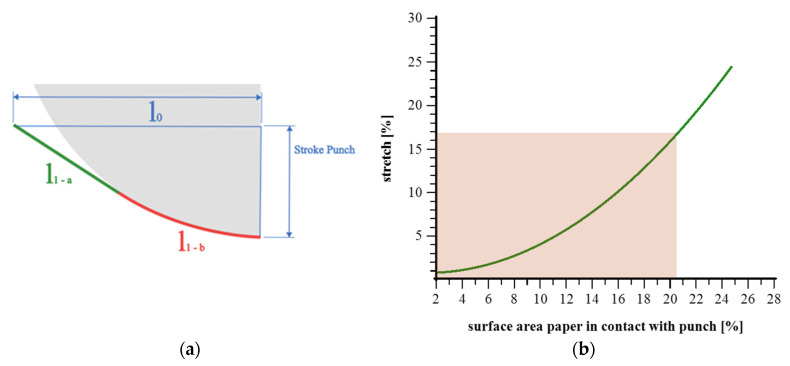
Deformation behavior of paper during forming: max stretch and contact area analysis. (**a**): Schematic representation of l_0_, l_1_, and the punch stroke; (**b**): relationship between the percentage of the paper surface in contact with the punch and the resulting stretch.

Contact with the heated punch evolves throughout the forming process. Initially, only the central upper region of the material contacts the punch. As forming progresses, the contact zone expands downward and outward. Toward the end of the stroke, the curved region l_1−b_ has a prolonged and direct contact with the heated punch surface, intensifying local thermal and mechanical effects. Meanwhile, the straight segment l_1−a_ has no contact and thus has a lower thermal exposure. Figure 5b shows the relationship between the percentage of the paper surface in contact with the punch and the resulting stretch. The figure illustrates a regression function, computed from data points with punch strokes spanning 1 mm to 14 mm, at 1 mm intervals. The shaded area marks the experimental range, where the maximum stretch reached aligns with the punch-contacted region identified in the first diagram.

### 3.1. Results on Material Moisture

**Figure 6:**
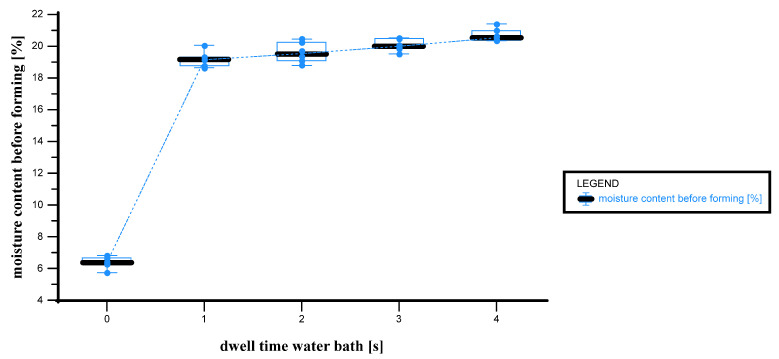
Box-plot–line graph dwell time in water bath vs. moisture content before forming. x axis: dwell time in water bath [s]; y axis: moisture content [%]; n = 30; I = 3.

Figure 6 illustrates the relationship between dwell time in the water bath and the moisture content before forming, expressed as a percentage.

At a dwell time of 0 s, the moisture content is at its lowest, with values ranging from 5.7% to 6.8%, with a median of 6.3%. As the dwell time increases to 1 s, there is a rapid and substantial increase in moisture content, reaching values between 18.4% and 20.0%, with a median of 19.3%. This sharp jump indicates rapid water absorption during initial immersion. At 2 s, the moisture content stabilizes somewhat, fluctuating between 18.8% and 21.4%, with a median of 20.2%. This suggests that the paper is nearing saturation. At 3 s, moisture levels remain high and consistent, ranging from 19.5% to 20.5%, with a median of 20.1%. At the longest dwell time of 4 s, the highest values are recorded, between 20.4% and 21.4%, with a median of 20.4%. This indicates that the material has largely reached its absorption capacity. The chart clearly shows that most moisture absorption occurs within the first second of water bath exposure. After this initial jump, moisture content plateaus, with only minor increases for longer immersion times.

This progression aligns well with the absorption stages described in [22] for automatic Cobb testing. It supports the view that absorption occurs in distinct phases—a rapid initial uptake, followed by a slower diffusion into the internal structure, and, finally, a saturation phase dominated by fiber swelling.

**Figure 7:**
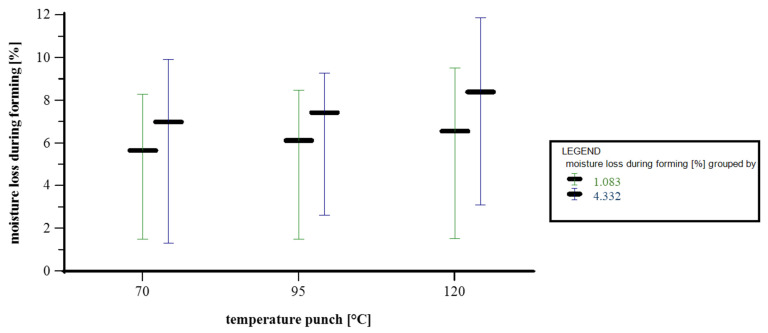
Interval plot depicting temperature punch vs. moisture loss during forming. x axis: temperature punch [°C]; y axis: moisture loss during forming [%]; grouped by contact time punch [s]; n = 30; I = 3.

Figure 7 illustrates how moisture loss in the formed area depends on the punch temperature (70 °C, 95 °C, or 120 °C), grouped by punch contact time (1.083 s in green and 4.332 s in blue). The moisture loss was converted to the area in contact with the punch (as described in Figure 3); drying by radiation is not considered within the scope of this study. At 70 °C, the mean moisture loss for a contact time of 1.083 s is 6.79% (range: 4.33–8.30%), while for 4.332 s, the mean is 8.19% (range: 1.29–9.92%). At 95 °C, the mean moisture loss is 7.19% at 1.083 s (range: 5.85–8.83%) and 8.18% at 4.332 s (range: 2.60–9.26%). At 120 °C, the moisture loss further increases, averaging 7.42% at 1.083 s (range: 5.75–9.51%) and reaching a mean of 8.95% at 4.332 s (range: 3.08–11.86%). Overall, the data show that moisture loss increases both with punch temperature and with longer contact time. The rise in mean values from 6.79% (70 °C, short contact) to 8.95% (120 °C, long contact) illustrates the combined effect of thermal input and mechanical interaction duration on water evaporation during forming. This trend aligns with heat conduction theory, whereby higher temperatures create greater thermal gradients and longer contact allows for more energy transfer into the paper [23].

Paper drying involves an initial heating phase, followed by constant-rate drying, where free water evaporates from the surface via capillary action. As free water depletes, the drying rate slows due to diffusion limitations and hygroscopic binding, eventually leading to negligible water removal [24]. Since thermal conductivity rises with both temperature and moisture content [24], heat transfer becomes more effective under hot, moist conditions. In summary, the observed increase in moisture loss reflects the combined effects of conduction and drying dynamics, both strongly influenced by thermal input and contact duration. However, it is important to note that although the moisture loss was normalized to the punch contact area at maximum stroke, the actual contact time across the surface is not uniform. As described above, the punch penetrates the material during forming, meaning it remains in contact with the paper longest at the deepest point of the cavity. This uneven contact distribution may help explain the considerable scatter observed in the data. Furthermore, the water is first removed via capillary flow, followed by slower mechanisms like surface diffusion and vapor transport as drying progresses [25,26]; therefore, the moisture distribution in the paper’s z-direction cannot be assumed to be homogeneous and cannot be measured reproducibly with the method used within these trials.

Fiber structure modification during drying is another point that is relevant to paper drying. Water evaporation reduces volume; gas fills some free space, while the remaining space collapses, leading to shrinkage. This shrinkage, describing volume, area, and length reduction, is non-ideal in anisotropic materials like paper, resulting in directional shrinkage [27]. Although only a small amount of water was removed, structural changes were still observed; specifically, samples that were dried more intensely exhibited waviness, a characteristic that is undesirable in packaging applications.

Concerning the method, it must be noted that moisture content was measured be-fore and after forming in separate runs using identical parameter settings, allowing for rapid data acquisition to reflect, as closely as possible, the moisture levels present during the actual forming process; however, sample variability may still introduce fluctuations and increase measurement error. Advanced in-line measurement systems using NIR or capacitive sensors may yield more precise and accurate data [16].

### 3.2. Results on Maximum Material Stretch

**Figure 8:**
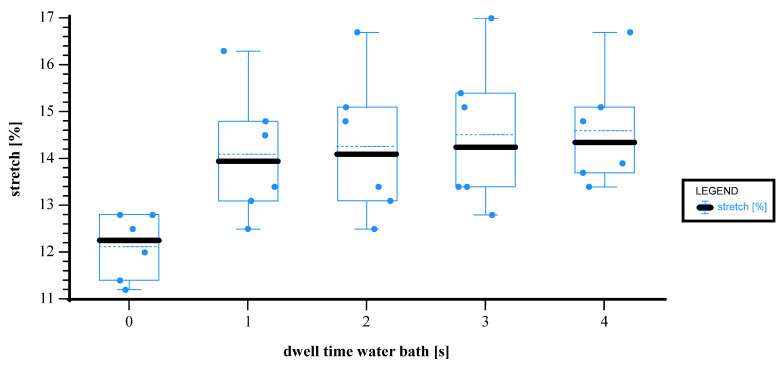
Box-plot–line graph for dwell time in water bath vs. stretch [%]. x axis: dwell time in water bath [s]; y axis: stretch [%]; n = 30; I = 3.

The chart in Figure 8 illustrates how the dwell time in the water bath affects the stretch [%] of the paper during the forming process. The calculated stretch levels derived from the punch stroke for each parameter setting are grouped by dwell time, ranging from 0 to 4 s. At a dwell time of 0 s, the minimum stretch is 11.2%, the maximum is 12.8%, and the median is 12.25%. When the dwell time increases to 1 s, the stretch improves remarkably. The minimum is 12.5%, the maximum is 16.3%, and the median is 13.95%. This indicates that even a short water exposure has a strong softening effect on paper. At 2 s, the minimum remains at 12.5%, while the maximum increases to 16.7%, with a median of 14.10%. For 3 s, the stretch values range from a minimum of 12.8% to a maximum of 17.0%, with a median of 14.25%. These values suggest that the material maintains a high stretchability level without much further gain. Finally, at 4 s, the minimum stretch is 13.4%, the maximum is 16.7%, and the median is 14.35%. These values confirm a plateau in stretch performance at longer dwell times. Prior research also links moisture content ranging from 10% to 20% with improved elongation [5,15]. For uncreped paper, the added moisture enhances flexibility, particularly in the cross direction (CD), but reduces tensile strength, especially in the machine direction (MD). This is due to the water weakening the fiber bonds and promoting plastic deformation [9]. The tooling used within this experiment did not allow for distinguishing between MD and CD behavior; however, the addition of moisture may facilitate a faster and more complete relaxation of the creped structure. The enhancement of flexibility due to added moisture is also evident from the reduced stretch values observed in the unmoistened samples. In these cases, the heated punch additionally dries the material, resulting in a lower stretch compared to the reference samples. However, the observation of a plateau in maximum stretch values indicates that paper humidification can only improve properties to a certain extent; once the material’s pores are saturated, over-humidification and subsequent fiber swelling may occur, leading to irreversible structural changes [22].

**Figure 9:**
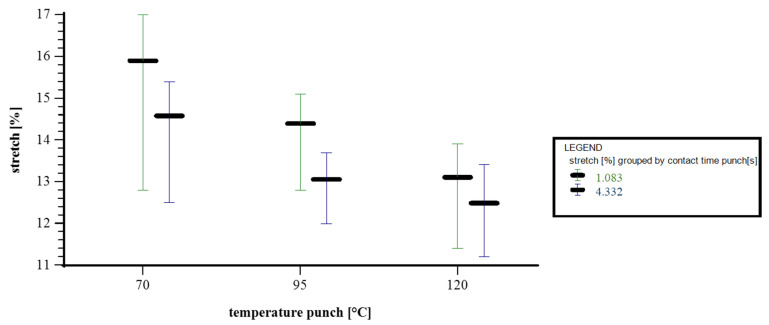
Interval plot depicting temperature punch vs. stretch. x axis: temperature punch [°C]; y axis: stretch [%]; grouped by contact time punch [s]; n = 30; I = 3.

Figure 9 illustrates how the stretch percentage of the paper material is affected by punch temperature (70 °C, 95 °C, and 120 °C), grouped according to the punch contact time (1.083 s and 4.332 s). At 70 °C, the highest stretch values are observed. For a contact time of 1.083 s, the stretch ranges from 12.8% to 17.0%, with an average of 15.7%. At a longer contact time of 4.332 s, the stretch decreases slightly, ranging between 12.5% and 15.4%, and averaging 14.45%. At a punch temperature of 95 °C, the stretch values are lower overall. For 1.083 s, the values range from 12.8% to 15.1%, with a mean of 14.3%. For 4.332 s, the stretch reduces further, with values between 12.0% and 13.4%, and an average of 12.9%. At the highest temperature of 120 °C, the stretch is at its lowest. The shorter contact time (1.083 s) yields a stretch between 11.4% and 13.4%, with an average of 12.9%. With 4.332 s of contact, the stretch drops slightly further to a range of 11.2% to 12.8%, and a mean value of 12.25%. In summary, the chart clearly demonstrates that stretch decreases with increasing punch temperature, and that longer contact times generally result in a lower stretch, particularly at higher temperatures.

In contrast to the findings of this study, which show a decrease in stretch with increasing punch temperature, earlier studies have reported a positive influence of elevated temperatures on the extensibility of paper [10], describing how temperature can soften the fiber structure, particularly of hemicellulose and lignin, by altering the moisture content and promoting polymer softening and glass transition processes. However, their experiments were conducted using fully dried and oil-impregnated samples, which excluded drying effects during heating—conditions that differ significantly from the present setup.

Similarly, Stotz et al. (2022) reported maximum stretch levels of up to 21% using the same paper grade in a comparable forming process. In their experiments, the paper was preheated homogenously across the entire sample area using a servo-driven heating plate prior to forming [28].

These contrasting results can be explained by key differences in experimental design. In the present study, heat is introduced only locally through a punch during the forming step, and the contact is not spatially or temporally uniform. As previously discussed, the punch contacts the material more extensively and for longer durations at the deepest point of the cavity, which likely results in uneven heating and drying.

**Figure 10:**
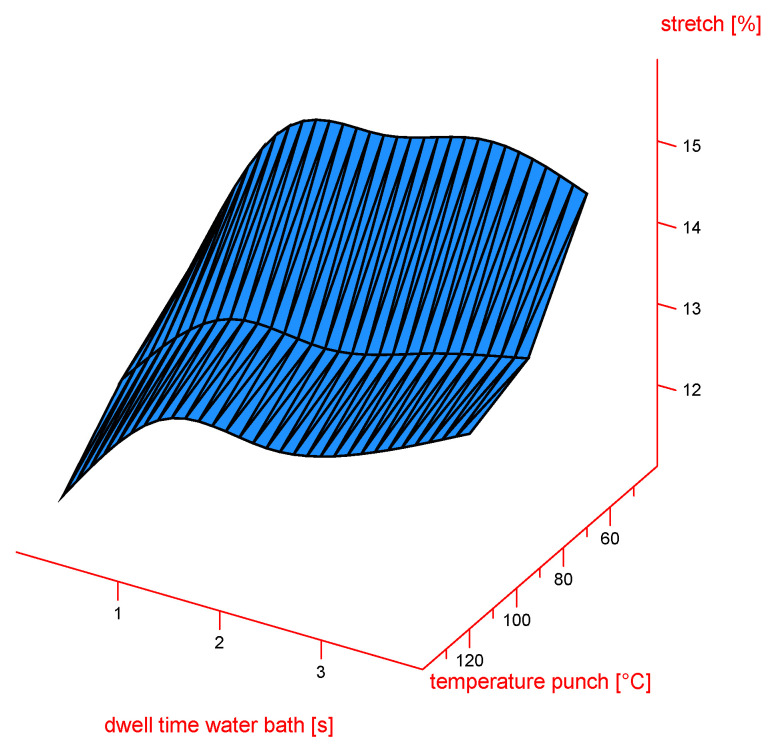
Surface plot depicting stretch [%]. x axis: temperature punch [°C]; y axis: dwell time in water bath [s]; z axis: stretch [%]; n = 30; I = 3.

The 3D surface plot in Figure 10 illustrates the influence of two process parameters—dwell time in the water bath (x axis) and punch temperature (y axis)—on the resulting stretch (%) of the material (z axis). The results show that increasing the dwell time in the water bath leads to an increase in the stretch. In contrast, higher punch temperatures result in a reduction in the stretch. Maximum stretch values are observed at long dwell times combined with lower punch temperatures.

A stepwise regression based on a cubic model is conducted using the software Camline Cornerstone (Version 8.1) using the data obtained from the experimental plan shown in Table 1. The term significance table is given in Table 2. The model shown is a multiple regression analysis that examines how various variables influence the target variables moisture content before forming, moisture loss during forming, and stretch [%].

Table 2 shows the term significance table for the model. The dwell time in the water bath [s] has a *p*-value of 8.5 × 10^−7^, indicating a highly significant effect in statistical terms. Likewise, the punch temperature [°C] shows an even stronger level of significance, with a *p*-value of 2.57 × 10^−10^, suggesting it plays a statistically robust role in the model. The punch contact time [s] is also statistically significant, with a *p*-value of 9.6 × 10^−6^, confirming its relevance in explaining the variation observed in the data. Furthermore, the squared term of the dwell time ([s]^2^), which captures potential nonlinearity in the model, is significant as well (*p* ≈ 0.00014). Taken together, these *p*-values confirm that each individual parameter contributes meaningfully to the model from a statistical perspective.

The model’s overall fit is also strong, with an R-squared value of 0.8946, indicating that about 89.5% of the variability in the dependent variable is explained by the included predictors. The adjusted R-squared of 0.8777 confirms that the model remains highly explanatory even when accounting for the number of predictors.

An optimization analysis was made with the model described. Table 3 shows the optimal process settings, i.e., a water bath dwell time of 3.03 s, a punch temperature of 70.00 °C, a contact time of 1.08 s, and a stretch of 16.50%. The overall desirability of this configuration is 0.456.

**Figure 11:**
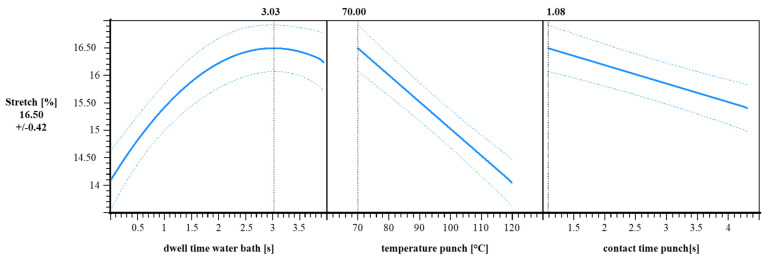
Response vs. predictors graph for forming trials for stretch [%]. x axis: dwell time in water bath [s], temperature punch [°C], contact time punch [s]; y axis: stretch [%]; n = 30; I = 3.

The diagram in Figure 11 presents the effects of three process parameters on material stretch [%], evaluated using a response surface model. The plots illustrate how dwell time in the water bath, punch temperature, and punch contact time influence the resulting stretch. The dashed lines represent confidence intervals, illustrating the model’s predictive uncertainty across the parameter ranges.

The relationship between dwell time and stretch exhibits a nonlinear, saturation-type trend. As the dwell time increases, the stretch rises sharply, reaching a maximum at approximately 3.03 s. This indicates that prolonged thermal exposure in the water bath enhances material deformability up to a certain point, beyond which further improvements are limited or even reversed.

In contrast, the influence of punch temperature shows a linear and negative trend. As the punch temperature increases from 70 °C to 120 °C, the stretch decreases nearly proportionally. This linear behavior suggests a consistent thermal effect on the material’s response, at least within the investigated temperature range.

A slightly decreasing and linear trend is also observed for the punch contact time. Although the slope is less steep than in the temperature plot, the stretch declines steadily as contact time increases.

The maximum predicted stretch is 16.50 % with a standard deviation of ± 0.42 %, which is achieved under the following optimized conditions: a water bath dwell time of 3.03 s, a punch temperature of 70.0 °C, and a punch contact time of 1.08 s.

**Figure 12:**
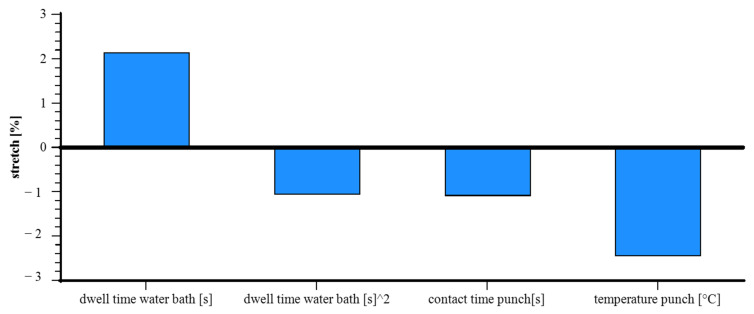
Pareto effects—influence of process parameters on the stretchability of paper: standardized effects based on regression analysis.

The bar chart in Figure 12 shows that dwell time in the water bath has the strongest positive effect on stretch, aligning with its high statistical significance (*p* ≈ 8.5 × 10^−7^). The squared term has a moderate negative effect (*p* ≈ 0.00014), indicating diminishing returns. Contact time and punch temperature both reduce stretch, with punch temperature having the strongest negative impact, matching its very high significance (*p* ≈ 2.57 × 10^−10^).

The results demonstrate that a uniformly elevated initial moisture level enhances the forming performance, yielding improved outcomes compared to unconditioned samples that were processed without temperature or moisture treatment. Conversely, forming under elevated temperatures without prior moistening led to the lowest stretch values observed. It should be noted that in certain industrial 3D paper forming processes, maximum material stretch is not the dominant factor and, within this study, no statement can be made on tribological effects or the influence on the compressibility of the wrinkles.

Overall, this study demonstrated an increase in maximum stretch of up to 3.5%. While temperatures below 70 °C were not investigated, exploring this range could be of interest, given the increased stretch behavior in comparison to the reference samples. Since key process parameters were recorded only in separate test runs, the implementation of more precise measurement technologies would be beneficial for identifying correlations more accurately and improving process stability.

Compared to other approaches, preconditioning with heat and moisture proved more effective than heating via the forming tool alone. These results underline the importance of how and when heat and moisture are applied. Further studies, such as radiative preheating [26], could deepen the understanding of process optimization.

## 4. Conclusions

Experimental investigations confirm that preconditioning paper via short water bath immersion significantly enhances its formability in 3D forming processes. A dwell time of 1 to 3 s results in rapid moisture uptake, reaching 18–21% moisture content and leads to an increase in maximum stretch of up to 3.5 percentage points compared to unconditioned reference samples.

A multiple regression analysis revealed that water bath dwell time has the strongest positive influence on material stretch (*p* ≈ 8.5 × 10^−7^), followed by statistically significant negative influences from punch temperature (*p* ≈ 2.57 × 10^−10^) and contact time (*p* ≈ 9.6 × 10^−6^). The resulting model explains approximately 89.5% of the variation (R^2^ = 0.8946). Optimal forming performance was achieved at a 3.03 s dwell time, 70 °C punch temperature, and 1.08 s contact time, yielding a predicted stretch of 16.5%.

Contrary to earlier studies reporting improved formability at elevated temperatures under constant moisture conditions [10,13], the localized heat input via the punch in this setup led to drying during the forming process and thus reduced stretch. This deviation from the literature can be attributed to the non-uniform thermal contact and in-process moisture loss. This is confirmed by the reduced stretch observed in unmoistened samples, caused by additional drying from the heated punch in contrast to the reference samples without preconditioning and forming without heat. Although temperatures below 70 °C were not examined, investigating this range may be worthwhile due to the increased stretch behavior compared to the reference samples.

These results underscore the dominant influence of moisture on paper extensibility, aligning with prior findings by Franke et al. (2018) [15], Östlund et al. (2011) [5], and Mark et al. (2001) [9]. Moisture reduces fiber stiffness by weakening hydrogen bonds and promoting plastic deformation.

In conclusion, integrating rapid and consistent moistening—such as water bath treatment—in high-speed packaging lines provides a practical and efficient method for improving forming behavior [16]. Future work should focus on in-line moisture and temperature measurement and the evaluation of alternative heating concepts like radiative homogenous preheating to enhance process control and repeatability [28].

## Figures and Tables

**Table 1:** Factors and factor levels for forming trials.

Factor	Factor Levels
Dwell time in water bath [s]	0
	1
	2
	3
	4
Temperature punch [°C]	set: 70 (measured on punch: 64.5)
	set: 95 (measured on punch:86.5)
	set: 120 (measured on punch: 109.8)
Cycle time [cycles/min] ≙ contact time punch [s]	10 cycles/min ≙ 4.332 s
	40 cycles/ min ≙ 1.083 s

**Table 2:** Term significance and coefficient table for forming trials.

Term	Coefficient	Significance
Constant	17.878	0
Dwell time in water bath [s]	1.600	8.538 × 10^−7^
Temperature punch [°C]	−0.049	2.574 × 10^−10^
Contact time punch [s]	−0.337	9.638 × 10^−6^
Dwell time in water bath [s]^2	−0.265	0.0001
R-Square	0.895	
Adj. R-Square	0.878	

**Table 3:** Optimization summary.

Term	Optimal
Dwell time in water bath [s]	3.027
Temperature punch [°C]	70.003
Contact time punch [s]	1.083
Stretch [%]	16.495
Desirability	0.456

## Data Availability

The raw data supporting the conclusions of this article will be made available by the authors on request.
